# Policy coherence for development as a potential framework for creating synergies between disaster risk reduction, climate change and food security: A theoretical review

**DOI:** 10.4102/jamba.v18i1.1837

**Published:** 2026-01-28

**Authors:** Samukelisiwe A. Myeni, Christo Coetzee, Leandri Kruger

**Affiliations:** 1 African Center for Disaster Studies, Faculty of Natural and Agricultural Sciences, North-West University, Potchefstroom, South Africa; 2 Unit for Environmental Sciences and Management, Faculty of Natural and Agricultural Sciences, North-West University, Potchefstroom, South Africa

**Keywords:** governance, policy coherence for development, disaster risk reduction, food security, climate change, Eswatini

## Abstract

This article examines the potential of policy coherence for development (PCD) as a framework for addressing the complex nexus between disaster risk reduction (DRR), climate change (CC) and food security (FS) in the context of Eswatini. While the interdependence of these domains has been recognised in theory and practice, policy responses in developing countries remain fragmented, resulting in duplication, inefficiency and weak implementation. Using an extensive literature review, the article conceptualises PCD and evaluates its capacity to provide horizontal and vertical policy integration, strengthen inter-ministerial and multi-stakeholder collaboration and align financial and operational mechanisms across governance systems. Five dimensions of policy coherence – conceptual, strategic, institutional, operational and financial – are employed to interrogate Eswatini’s fragmented governance landscape, where more than a dozen policies and frameworks exist but fail to translate into coordinated outcomes in managing the DRR, CC and FS nexus. In Eswatini, where over 70% of the population relies on subsistence agriculture and recurrent drought exacerbates poverty and food insecurity, embedding PCD principles into policy-making structures could bridge the gap between ambitious developmental agendas and local implementation realities.

**Contribution:**

The study concludes that institutionalising PCD can enhance resilience by integrating DRR, CC and FS policies, fostering joint accountability among a wide array of societal role players and enabling more efficient resource allocation. By moving from conceptual recognition to practical implementation, PCD provides a pathway towards sustainable development and improved governance of the DRR, CC and FS nexus in Eswatini.

## Introduction

Africa faces many challenges relating to climate change (CC), disaster risk and food insecurity (Valente et al. 2022). To this effect, disaster impact and losses associated with extreme climatic events have continued to overwhelm the continent and individual countries’ governance structures. Literature indicates that development outcomes are influenced by disaster vulnerabilities, climatic exposures and a lack of social, economic and political capacity (Biswas & Nautiyal 2023). According to Ahrens and Rudolph (2006), developing countries were characterised as lacking the capacity to cope with disasters on an administrative, organisational, financial and political level. It is the responsibility of government to manage disaster risk by creating an institutional arrangement that can build the capacity of social actors and individuals (Shah et al. 2020). In doing so, resilience can be measured based on the ability of social actors and an individual’s capacity to respond to and recover from disaster and climatic impact they have experienced (Aroca-Jiménez et al. 2025). Ahrens and Rudolph (2006) further quantify that 11% of people exposed to hazards live in countries with low economic development. Underdevelopment and susceptibility to disasters are closely related in that disasters cause loss of capital assets and disrupt production, including the flow of goods and services in the economy (Ahrens & Rudolph 2006). Biswas and Nautiyal (2023) further explain that countries within Africa have pre-existing critical challenges such as poverty, hunger, pandemics, environmental degradation and underdevelopment that are exacerbated by disaster impacts. This status quo necessitates the adoption of novel approaches to sustainable development that can foster greater integration between development sectors to ensure more significant disaster mitigation and resilience building (Surianto et al. 2019).

One such avenue is presented by policy coherence for development (PCD). Policy coherence for development is ideally placed in fostering the proactive management of complex developmental issues across various developmental sectors. Shawoo et al. (2023) elaborate that policy coherence is widely seen as critical to the successful implementation of global policy frameworks, with many arguing that coherent policy-making will help governments to navigate trade-offs between developmental and divergent development pathways. Within this framework, conceptual coherence ensures actors across sectors share common understandings of resilience, vulnerability and adaptation; strategic coherence promotes the articulation of these concepts within national and departmental frameworks; institutional coherence establishes mechanisms such as inter-ministerial committees or task teams to overcome silos; operational coherence facilitates coordinated planning and joint implementation; and financial coherence aligns budgetary processes to support integrated action. Collectively, these dimensions of policy coherence underscore how PCD can provide a structured pathway for integration across governance systems to facilitate the improved management of the disaster risk reduction (DRR), food security (FS) and CC nexus in a developing country context, especially those such as Eswatini where subsistence agriculture secures the lives and livelihoods of significant parts of the population (Habiba, Abedin & Shaw 2016).

The purpose of the article is therefore to investigate PCD as a potential avenue for managing complex developmental problems related to FS, DRR and CC. Specifically, the article conceptualises PCD and unpacks its key characteristics and application, ensuring greater integration between these priorities, paying particular attention to how the five levels of policy coherence can be mobilised through PCD to address Eswatini’s fragmented governance landscape as it relates to the nexus. By doing so, the article endeavours to highlight the opportunities for adopting PCD in Africa but also engages critically with the challenges of adapting what is originally a European framework to an African context.

### Nexus between disaster risk reduction, climate change and food security in Eswatini

The Sustainable Development Goals (SDGs) emphasise the reduction of poverty, hunger and food insecurity as prerequisites for a country’s economic development (Shah 2022). The realisation of the SDGs does not occur in isolation, as various conditions – such as disasters, climate variability and socio-economic factors – can hinder their achievement. For example, households exposed to disaster shocks such as floods and droughts are more likely to experience food insecurity compared to those that do not face such events (Dlamini 2025). Mamba et al. (2025) identify a direct link between disasters, CC, poverty and food insecurity, noting that hazards alter the fiscal landscape and hinder access to goods, service delivery and livelihoods for households and communities.

In Eswatini, the impact of hazards such as droughts, heatwaves, wildfires, floods and storms is prominent and remains an ongoing challenge (Mkhatshwa et al. 2024). Drought, in particular, is a recurrent challenge, affecting approximately 14% of the population annually (Mkhatshwa 2024). This has a direct impact on food availability, resulting in reduced affordability and access to nutritious food (Atanga & Tankpa 2021; Han, Keeni & Fuyuki 2024). Climate change and disaster risk affect food production by reducing crop yields and livestock production, upon which many Eswatini households rely. Specifically, 70% of the population depends on subsistence farming for both livelihood generation and food supply. Food insecurity, poverty and poor nutritional outcomes become chronic when subsistence farming is disrupted by environmental impacts (Rother et al. 2022). Therefore, DRR, CC and FS are deeply interconnected, and using a nexus approach enhances their understanding and strengthens the research base.

The nexus between CC, FS and DRR should be managed in a coherent way to limit the impact of adverse weather on food production, access and availability (Ali et al. 2025). Spinazzola and Cavalli (2022) add that Agenda 2030 also encourages the integrated management of interrelated development issues such as CC, FS and DRR. For example, SDG Target 17.14 advocates for policy coherence and integration across governance sectors to promote sustainability. As such, the UNDRR (2022:11–14) argued that for policies that aim to address complex developmental problems such as the CC, FS and DRR nexus to be effective, policy coherence should be pursued at five different levels, that is, conceptual, strategic, institutional, operational and financial levels. *Conceptual coherence* relates to whether various interrelated government departments (i.e. that share an associated developmental mandate) understand the theoretical necessity for integrating their activities but also have a shared understanding of basic concepts such as resilience, adaptation and vulnerability. Once conceptual integration is achieved, key national and departmental policies should clearly articulate the need to manage the nexus in an integrated manner. Through this, *strategic coherence* of the nexus is achieved. To move conceptual and strategic integration into the realm of implementation, it is important to facilitate *institutional coherence*. Institutions such as inter- and intra-governmental task teams and committees need to be established to ensure overlaps in mandate are eliminated and political buy-in is assured. These institutions can also serve as the focus for *operational coherence*, as they can facilitate coordinated planning, development of shared implementation frameworks and methodologies and integrated resource allocation across various departments and levels of government. Finally, countries should endeavour for greater *financial coherence* by outlining clear funding strategies that detail budget allocation from various government departments for the integrated management of the CC, FS and DRR nexus. Additionally, funding strategies should outline potential budgeting shortfalls and identify private sectors and donors that could help to fill funding gaps.

A failure to address this nexus coherently disproportionately affects vulnerable communities with limited resources (Rother et al. 2022). This is particularly relevant to recurring droughts in Eswatini, which are weather-related disasters characterised by erratic rainfall and dry spells (Mamba 2019; Mlenga & Jordaan 2019). Currently, more than 16 policies, legislation, strategies and frameworks exist in Eswatini to address individual aspects of the CC, FS and DRR nexus. Despite Eswatini’s governance and policy development efforts, the implementation of policy goals remains fragmented on intergovernmental and intradepartmental levels, meaning the nexus is not addressed in a streamlined or comprehensive manner. This indicates that although the current legislative and policy frameworks are promising, they potentially do not adhere to various levels of policy coherence outlined above and require further development (Stein & Jaspersen 2019).

As identified, governance gaps in Eswatini require that policy and literature analyses be holistic, drawing on diverse conceptual frameworks. Frameworks that inform decision-making and support PCD include the institutional analysis and development (IAD) framework, value chain analysis, actor-network theory and strategic risk frameworks. Among these, IAD and value chain analysis are most useful in shaping progressive organisational direction, particularly in an Eswatini setup. According to Ostrom (2011), policymakers and stakeholders should adopt the IAD framework to operationalise PCD. The IAD approach promotes dissecting institutional components to clarify policy scopes and desired outcomes. In Eswatini, the policy challenges at the nexus of DRR, CC and FS include dependency on humanitarian aid and rising poverty (Vulnerability Assessment and Analysis [VAA] 2024 coherence for development offers a pathway forward by addressing these root causes while promoting strategic improvements for resilience and sustainable development.

The value chain analysis framework helps guide policymakers and implementers on integrated strategies to enhance climate resilience, reduce disaster risk and mitigate food insecurity. Kaplinsky and Morris (2001) define this approach as one that maps end-to-end activities and identifies transformation strategies for improved coordination. According to Villamayor-Tomas et al. (2015), there is a causal relationship between climate instability, disasters and agriculture. Because agriculture directly influences food availability, access, utilisation and stability (Pawlak & Kołodziejczak 2020), the sector plays a critical role in ensuring FS and building climate and disaster resilience. Muñoz et al. (2025) argue that resilience-oriented policy development must address existing community vulnerabilities through strategies that integrate CC, FS and DRR. Applying a value chain approach in Eswatini could aid governance actors in managing risks and developing adaptive plans to address poverty and hunger.

In conclusion, policy coherence emphasises joint planning by various sectors and stakeholders. According to Yamazaki-Honda (2024), the challenges posed by DRR, CC and food insecurity must be tackled through a coherent, risk-informed policy-making system to achieve a resilient and sustainable society. Therefore, the Government of Eswatini should explore innovative approaches such as PCD to manage the CC–FS–DRR nexus in order to overcome challenges for sustainable development.

### Origins and conceptualisation of policy coherence for development

The global development community has long considered the advantages of having an integrated approach to development policy. Policy coherence for development is a concept that has emerged in recent decades. In 1991, PCD emerged in the Development Assistance Committee (DAC) of the Organisation for Economic Co-operation and Development (OECD). The committee recommended that policy issues related to sustainable development should integrate into one another and not challenge or contradict existing policies (OECD 2010). In 1992, PCD came to prominence in the European Union (EU) (Barry & Mathews 2010; Righettini & Lizzi 2021). The PCD agenda was first established by the Maastricht Treaty in 1993 and was reinforced in 2009 by the Treaty of Lisbon (Barry & Mathews 2010; Bocquillon 2018; Nilsson et al. 2012; Righettini & Lizzi 2021; Selianko & Lenschow 2015). In article 30.2(d) of the Maastricht Treaty, a call was made to the EU to adopt consistency between policies by adopting a single institutional framework for the governance of developmental issues. The Treaty of Lisbon reinforces and extends PCD’s obligation to the whole EU. Specifically, in article 208, the Treaty advocates for applying PCD principles to streamline development cooperation among countries and foster integrated development policies within the EU. Koff (2016) elaborates that through these treaties, the EU has prioritised five developmental areas, namely, (1) trade and finance, (2) CC, (3) global FS, (4) migration and (5) security. The growth of the term since its origin has encouraged EU policymakers to recognise coherence and integration in policy-making as critical features of development and the achievement of the 2030 Agenda of Governance (Righettini & Lizzi 2021; Zeigermann & Böcher 2019).

The 2030 Agenda represents a milestone for sustainable development and governance in that it encourages both the international and national community to accept comprehensive, binding set goals and targets. However, achieving the 17 SDGs that are outlined in the 2030 Agenda is a challenge and requires new and transformative approaches to policy development, such as PCD, to be implemented (Righettini & Lizzi 2021; Zeigermann & Böcher 2019). An integrated approach to development also fosters greater cooperation among and across various policy sectors (Nilsson et al. 2012). The need for great policy integration is also recognised in the SDGs themselves. Specifically, SDG 17.14 calls for the ‘enhancement of policy coherence for sustainable development’ within governance (United Nations Environment Programme [UNEP] 2023). Accordingly, the central goal is that no one goal can be achieved without another goal. This interdependence between SDGs necessitates political actors and policymakers adopting integrated solutions towards the achievement of SDGs and overcoming certain trade-offs between the developmental goals. For example, SDG 2 and 6 present an opportunity where developmental issues relating to food and water would need to be addressed synchronously. In this instance, SDG 2 addresses sustainable agricultural practices that alleviate hunger and poverty, while SDG 6 addresses water usage and provision of clean water for all. Consequently, these two SDGs need to be closely integrated at the policy and project implementation levels to avoid mutually adverse outcomes and achieve sustainable development in the agricultural sector. To manage such trade-offs, developmental policies being made should take a holistic approach and strengthen governance, social, environmental and financial systems in an integrated fashion at different spatial scales (Bogers et al. 2022). Focusing on developmental issues in their full complexity necessitates the development of policy mechanisms that will contribute to the complete integration and accurate implementation of development policies and programmes (Zeigermann & Böcher 2019).

Policy coherence for development provides a mechanism that integrates all the internationally agreed-upon targets of SDGs and allows countries to break away from policy silos and achieve synergies in actions to be done to effectively govern (Yunita et al. 2022). Biermann, Kanie and Kim (2017) concur by describing the SDGs as a comprehensive and true reflection of the integrating global goals, global development and environmental sustainability in governance agendas. Given this casting, PCD emerges as a priority to effect development and encourage mutually reinforcing policies (Yunita et al. 2022). To gain a deeper understanding of PCD, the concept thereof needs to be clarified, which will follow next.

### Defining policy coherence for development

Various definitions of PCD emerged from literature. A founding definition is provided by the OECD, which describes PCD as the process that considers trade-offs and potential synergies across key developmental areas such as trade, investment, agriculture, health, education and the environment to support efforts to achieve the internationally agreed development goals (OECD 2001). This definition establishes one of the core tenets of PCD, which is that effective development policies are not isolated issues but rather interconnected, thereby addressing different policy objectives in unison (Nilsson et al. 2012). Carbone and Keijzer (2016) in turn define the concept of PCD as the synergy and interaction between foreign aid and all other development-related policy. This definition continues to stress the need for unity between policies at all levels, including between those of state and non-state actors. According to Barry, King and Matthews (2010:2), PCD occurs ‘when policies addressing a wide range of domestic issues are in support and align with international development issues’.

As a result of these various definitions, this article argues that PCD can be defined as the process by which governments should interlink developmental sectors and policy areas at different spatial scales to achieve integrated governmental policies that address complex sustainable development issues. Arguably, the historic lack of coordination in the formulation and implementation of development policies across sectors and levels of government has created barriers, including a lack of buy-in, conflicting mandates and misallocation of limited resources in many developing countries (Curran et al. 2018). The PCD approach ensures that policies prioritise inclusiveness between all developmental sectors, including FS, CC and DRR (Meuleman 2018). The next section will outline the pillars and building blocks of PCD in more detail.

### Pillars and building blocks of policy coherence for sustainable development

In 2019, the OECD introduced recommendations on policy coherence for sustainable development as underpinning guidelines for PCD (OECD 2019). To enhance PCD, governments must consistently design and implement coherent policies that can be used to streamline administration, planning, policy review, budgeting and regulatory development. To this effect, the OECD (2021) introduced three overarching pillars to enhance PCD that include:

- A strategic vision for implementing the 2030 Agenda underpinned by a clear political commitment and leadership to enhance policy coherence for sustainable development.
- Effective and inclusive institutional and governance mechanisms to address policy interactions across sectors and align actions between levels of government.
- A set of responsive and adaptive tools to anticipate, assess and address domestic, transboundary and long-term impacts of policies.

The main goal of the three pillars is to establish a comprehensive standard to help countries equip policymakers and key stakeholders with the necessary institutional mechanisms and policy tools to enhance policy coherence and address integrated economic, social and environmental goals while accelerating progress toward the SDGs (OECD 2021). In addition to the three pillars, eight building blocks were identified to enhance countries’ ability to achieve the above pillars. The eight building blocks were envisioned to illustrate how different institutional mechanisms fit together and can contribute towards building higher degrees of policy coherence. These building blocks and their interaction with the three principles of PCD are outlined in this section.

The OECD (2017) conceptualises policy coherence for sustainable development through a cycle involving eight building blocks. This interaction illustrates how institutional structures and mechanisms operate to ensure a coherent implementation of SDGs. These building blocks illustrate a holistic approach involving mobilising whole-of-government action; balancing economic, environmental and social concerns; reconciling short- and long-term priorities; addressing potential negative impacts of domestic policies beyond borders; ensuring coordinated and mutually supporting efforts across sectors; involving sub-national and local levels of government; engaging key stakeholders beyond government; and using monitoring and reporting systems to inform coherent policy-making.

**FIGURE 1:**
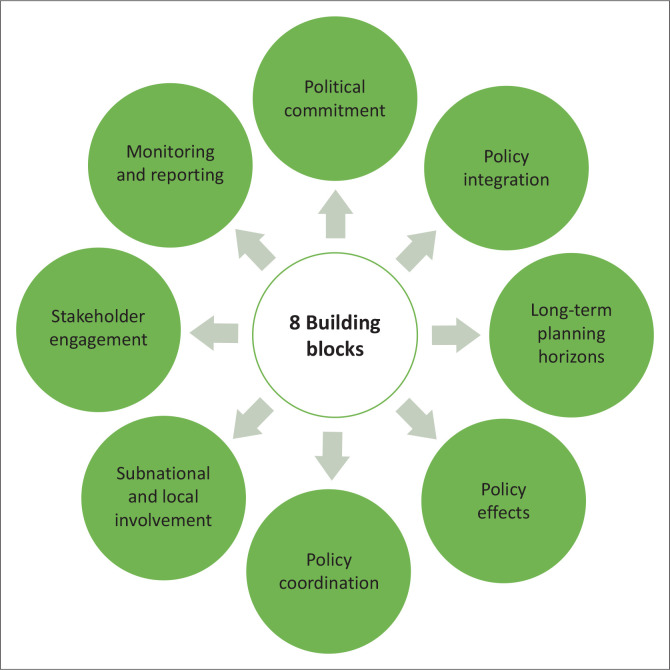
Eight building blocks of policy coherence for development.

The eight building blocks, as illustrated in Figure 1, include (1) political commitment; (2) policy integration; (3) long-term planning horizon; (4) policy effects; (5) policy coordination; (6) sub-national and local involvement; (7) stakeholder engagement; and (8) monitoring and reporting. Using the principles of IAD, the study will explore the application of the PCD framework in the context of Eswatini. All components within each of the above-mentioned eight pillars will be dissected as a means of enforcing the value of PCD adoption towards the achievement of sustainable development.

### Building blocks related to Pillar 1 of policy coherence for development

The first three building blocks endeavour to support the achievement of Pillar 1, that is, a strategic vision for implementing the 2030 Agenda underpinned by a clear political commitment and leadership to enhance policy coherence for sustainable development.

#### Political commitment and leadership

Political commitment and leadership can be defined as the intentional buy-in and perpetual support by politically influential entities or individuals of activities that proactively address developmental problems facing a society (Baker et al. 2018:2). The OECD (2017) adds that political commitment is important to address complex developmental problems such as the CC, FS and DRR nexus, as it drives policy development, resource allocation (e.g. human and financial) and the creation of institutions between and within line ministries that aid in translating legislative commitments into concrete programmes at local, regional, national and international levels. Importantly, Nilsson et al. (2012) highlight that political commitment does not only stop with the formulation of developmental roadmaps and strategies but crucially has to extend to ensuring synergies and consistencies between disparate developmental policies and government departments and to project implementation and completion. Currently, it could be said that political commitment and leadership in Eswatini are mostly visible in the wealth of policy frameworks, legislation and strategic action plans to address developmental problems. For instance, currently, there are more than 16 policies, legislation and strategic frameworks and plans that seek to address drought mitigation in Eswatini. However, political commitment in the country seems to falter at the interface between policy formulation and implementation. Specifically, there is a severe lack of centralised coordination between different government departments and agencies working on issues relating to CC, FS and DRR, which often leads to the duplication of programmes across departments, inefficient use of limited financial and human resources and ineffective programme implementation to address the problem. Mobilising government departments to break free of their silos and to work together to address the CC, FS and DRR nexus remain an area that needs to be strengthened in the country. Jeroen and Candel (2019) argues that following a PCD approach could assist Eswatini in addressing the shortfall of political commitment at the level of implementation by clarifying roles, responsibilities, priorities and mechanisms that will facilitate coherent policies and cooperation between government departments and levels of government. Importantly, a PCD approach prioritises the focus on governmental partnership and joint responsibility for project implementation that addresses complex developmental problems. Cooperation fostered through the PCD approach is crucial in achieving the shared goal of resilience building and sustainable development between the FS, CC and DRR sectors in the country.

#### Policy integration

Policy integration focuses on capitalising on synergies and managing trade-offs between economic, social and environmental policy areas, as well as ensuring consistency with internationally agreed-upon goals (Jeroen & Candel 2019; Trein et al. 2021). Howlett, Vince and Del Río (2017) add that policy integration within this building block entails stepping away from an individualistic governance system and adding cross-sectoral linkages within policies, mandates and frameworks (OECD 2018). It is observed by the OECD that the principle of policy integration is best promoted by the presence of inter-ministerial mechanisms that foster policy integration and mainstreaming and incorporate SDGs into the work of all line ministries (Trein et al. 2021). Currently, intergovernmental mechanisms for integrated governance in DRR, CC and FS in Eswatini are not properly developed at both horizontal and vertical levels of governance (Phungwayo, Kushitor & Koornhof 2021). As a consequence, the DRR, CC and FS nexus is still very much managed in government silos that duplicate actions, lack broad stakeholder participation, use resources inefficiently and inevitably fail to achieve the long-term strategic developmental objectives of the Government (Phungwayo 2021). Government departments working in silos also hamper the sustained funding of CC, FS and DRR projects and hinder the building of political support for long-term project implementation.

To address the current lack of policy integration in managing the FS, CC and DRR nexus in Eswatini, PCD can be beneficial in informing the development of inter- and intra-governmental governance mechanisms, for example, advisory forum on the FS, CC and DRR nexus (Aiyede & Muganda 2023). Such forums could consolidate opinions, views and inputs of various groups regarding problems, challenges, goals, policies and optimal solutions in managing the FS, CC and DRR nexus based on collective consensus (Mkhatshwa 2024). Improved collective action brought about by the adoption of PCD will aid in building the resilience of Eswatini to disaster risk, CC and food insecurity (Morton, Pencheon & Squires 2017).

#### Strategic long-term horizon planning

Strategic planning with a long-term focus on the establishment and development of legislation, policies and strategic action plans that clearly stipulate development goals and activities ranging from 5-, 10- and 20-year cycles (Koff, Challenger & Portillo 2020). The principles that support this building block include building progressive, strong and inclusive political commitment and leadership at the highest political level and fostering a holistic approach to governance at a variety of spatial and temporal scales (Peters 2015; Trein et al. 2021). The key to achieving this building block is to promote and enhance priority analysis, strategic planning and public information sharing at all levels of government.

In Eswatini, the country’s short-, medium- and long-term development goals are pursued through various tools, policies and government commitments. In the short term, in 2019, the Kingdom of Eswatini developed a Strategic Road Map (2019–2022) that aimed to establish a policy framework to ensure sustainable economic development, financial stability and growth while improving the quality of life in Eswatini. Following that, the country developed a medium-term development policy called the Eswatini Strategic Plan (2020–2025), which guides the development and economic recovery by recognising the critical dimensions of human development, including poverty reduction, employment creation, gender equity and social integration. In the longer term, Eswatini is a signatory country to the SDGs, which implies that the country has endeavoured to integrate and achieve aspects of the SDGs in all developmental policies by 2030.

Despite this commitment towards the facilitation of policy integration within the existing short-, medium- and long-term developmental plans, implementation problems persist in Eswatini. According to Nemakonde, Van Niekerk and Becker (2021), policy commitments made by the government of Eswatini are not always supported by existing legal mandates that establish the roles and duties of various government departments. Specifically, individual government departments such as Agriculture, the Ministry of Environmental and Tourism Affairs and the Prime Minister’s office are given the sole mandate by law to be responsible for the management of either DRR, CC or FS. The need to cooperate with other departments in managing their primary task is not always clearly stipulated, or a clear direction given on how to achieve it by legislation. This therefore makes policymakers and decision-makers reluctant to coordinate their functions and share resources with other departments working within the DRR, CC and FS nexus space. The silo approach to governance continues to be the main reason for the lack of achievement of long-term developmental goals in the country, as there is an inability to make collective decisions that will resolve complex developmental issues such as those presented by the DRR, CC and FS nexus (Scott & Ting 2021). Introducing a PCD approach to developmental governance in Eswatini will allow the government to enhance monitoring and evaluation mechanisms to include measures relating to the extent of interdepartmental cooperation as a measure of the efficacy of programme implementation (see Pillar 3 below). This could aid in the early identification of developmental interdepartmental silos and subsequent programme weaknesses, which can be addressed through targeted interventions that allow for realignment with the long-term development goals of the country.

On reviewing all three building blocks that form part of this pillar through the lens of the five categories of policy coherence as stated under the policy effects section of the article the achievement of this pillar in Eswatini mostly functions at the level of conceptual coherence. Specifically, most of the formulated policies recognise the key concepts such as resilience, FS, CC and coordination. However, conceptual understanding does not translate over to strategic coherence, with numerous policies failing to translate into integrated implementation. Institutional and operational coherence is also largely absent, as much of the governance of the CC, FS and DRR nexus is still being managed in silos, resulting in a lack of political buy-in across departments and different levels of government and duplication of programmes and inefficient use of resources. Financial coherence is also severely lacking, as there are no apparent cross-sectoral funding strategies or integrated budgetary frameworks in place to support the integrated management of the nexus.

The following building blocks to be discussed are related to Pillar 2.

### Building blocks related to Pillar 2

The next three building blocks endeavour to support the achievement of Pillar 2, that is, effective and inclusive institutional and governance mechanisms to address policy interactions across sectors and align actions between levels of government. The first building block to be discussed here relates to policy coordination.

#### Policy coordination

According to Ghymers (2005), policy coordination is an activity that enables dialogue and consensus between policymakers to agree to and act according to certain rules, goals and outcomes. The OECD (2018) adds that policy coordination is a crucial activity in any governance activity, as it streamlines policy implementation, how human and financial resources are allocated and the clarification of responsibilities to key role players. Proper policy coordination within government enables the identification and mitigation of divergences in policy implementation that lead to poor service delivery and situations where societal needs are not met (Herrera-Medina & Font 2023).

The existing legal and policy framework in Eswatini aims to integrate DRR and CC mitigation, adaptation and resilience strengthening within the agricultural and FS space (Disaster Management Act 2006). Dlamini (2025) elaborates that although the CC, FS and DRR nexus is mainstreamed into Eswatini’s development policies documentation, this integration does not translate to policy and project implementation. A potential reason for this situation is that in Eswatini, there are various sectors and stakeholders entrusted with carrying out the mandate of CC, FS and DRR. For instance, the Deputy Prime Minister’s Office is responsible for disaster risk issues; the Ministry of Environmental and Tourism Affairs is responsible for CC issues; and FS issues are mandated to the Ministry of Agriculture. However, a glaring challenge that exists is that there is no single entity responsible for coordinating the activities among various stakeholders (Swaziland Resilience Strategy and Action Plan [SRSAP] 2017). This poses a challenge to the achievement of an integrated approach to the management of the CC, FS and DRR nexus in the country, as no single entity takes responsibility nor does a mechanism exist to hold the others accountable for strengthening partnerships and networks, developing competencies, stakeholder participation and coordination to achieve the collaborative development vision and plans associated with the nexus.

The policy coordination building block within the PCD could assist in resolving divergences between sectoral priorities and policies while ensuring mutually supporting efforts across sectors and institutions in managing the CC, FS and DRR nexus (Bogers et al. 2022; Milton 2011). For instance, this building block can be implemented by introducing a coordination framework in Eswatini, as currently there are no coordination mechanisms or frameworks focused on the management of the CC, FS and DRR nexus. Developing a coordination framework could contribute to facilitating greater policy cohesion and cooperation between different government departments towards successfully achieving the country’s developmental goals and milestones (Chikezie et al. 2024).

#### Sub-national and local involvement

Sub-national and local involvement broadly refers to enabling the participation of regions, cities and municipalities in policy-making (Nonet & Van Tulder 2022). According to Koff and Häbel (2022) and Mensah, Mensah and Mensah (2022), this building block relates to the principle of the ‘leave no one behind’ approach as advocated by the SDGs. Consequently, sub-national levels of government are seen as crucial actors in ensuring that developmental programming reaches the most remote and vulnerable people in a society. Bowen et al. (2017) elaborate that the involvement of sub-national and local governments in governance ensures that a conducive and enabling environment for collective action, coherent policy-making, decision-making, implementation and monitoring of the use of SDGs is maintained throughout all spheres of government. The central aim is, therefore, to ensure that decisions made at the national level cascade down to local government for implementation (OECD 2018). The current governance structure in Eswatini makes provision for the involvement of sub-national and local government in the management of complex developmental issues. For example, in Eswatini, government consists of the national level (central head office), the sub-national level and Tinkhundla (constituencies), which constitute the local level. Decision-making, policies and strategies are made and formulated at the national level (central government). Once formulated, implementation is delegated to the sub-national and eventually local level. This decentralised governance should ensure that, despite the complexities of cascading decisions, an integrated approach is adopted to developmental policy at different levels of government.

In theory, the ideal of decentralisation is to bring government closer to the people; however, the coordinated management and implementation of complex developmental challenges such as the DRR, CC and FS nexus presents a huge challenge, especially for the Ministry of Tinkhundla and Administration (MTAD) (Nemakonde et al. 2021). Specifically, the Tinkhundla level of governance in Eswatini forms part of a parallel traditional governance structure, which primarily reports to local chiefs and eventually the monarch, rather than official government structures. Consequently, even if the government of Eswatini commits to managing DRR, CC and FS in an integrated manner, if this is not a priority or is not well understood by the parallel traditional system of governance, these policy commitments are unlikely to translate to local action and implementation.

By adopting the PCD approach, the government of Eswatini could emphasise the development of an intergovernmental, multi-stakeholder governance mechanism exclusively for the management of the CC, FS and DRR nexus. This mechanism should consist of selected chiefs or royally appointed focal points to represent the interests of the Tinkhundla level of governance as well as national government departments, the private sector and representatives. A multi-stakeholder governance mechanism could address some of the disjointed policy development and project implementation at all levels by enabling broad stakeholder involvement and coordination, joint policy-making and facilitating political will and understanding for the need to manage the CC, FS and DRR nexus in an integrated fashion at all levels of governance in Eswatini.

The following section will discuss the fifth building block, that is, stakeholder involvement.

#### Stakeholder engagement

Stakeholder engagement can be defined as the ‘practices that the organisation undertakes to involve stakeholders positively in organisational activities’ (Greenwood 2007:318). According to Castro (2022), inclusive engagement is an essential pillar for policies and decision-making in governance, as it ensures cross-cutting and complex developmental issues are critically discussed, accepted and adopted. Stakeholder engagement is also vital for understanding donor priorities, service delivery demands of communities and strengthening accountability within government (Castro 2022). Stakeholder engagement is a crucial activity that needs to be conducted to achieve desired developmental outcomes, as the diverse perspectives and resources of different stakeholders can aid in developing and implementing more successful and sustainable developmental programming (Meuleman 2018). The crux of having multiple stakeholders is associated with linking and building synergies between partners aiming to achieve the same goal (Greenwood 2007). These synergies ensure that there is a comprehensive strategic direction and operational excellence in governance towards the achievement of sustainable development for a better society (Phungwayo et al. 2021; Re & Magnani 2023; Unerman, Bebbington & O’Dwyer 2010). Nonet, Gössling and Van Tulder (2022) suggest that stakeholder engagement activities should be informed by an exhaustive list of stakeholders, including big and small corporations, governments, civil society groups, universities, communities and other organisations participating in the management of developmental problems such as the CC, FS and DRR nexus.

In Eswatini, the national government strives towards multi-sectoral governance in addressing developmental issues faced by the nation (Phungwayo 2021). However, Manyatsi and Mhazo (2014) observe that despite these efforts, challenges with stakeholder engagement in the management of cross-sectoral developmental issues (e.g. CC, FS and DRR) contribute to a lack of policy coherence. For instance, to reduce the burden of food insecurity in Eswatini, the government has implemented social grants. This initiative has had a positive impact on some food-insecure communities; however, it is inadequately coordinated, administered and implemented within a single ministry (Deputy Prime Minister’s Office), with limited input from other ministries or stakeholders such as the Ministry of Agriculture and the Ministry of Tourism and Environmental Affairs. Having a single ministry administer this task has often led to gaps in identifying the most vulnerable communities and identification programme beneficiaries and delays in enrolling beneficiaries in programme activities, thereby eroding the efficiency and effectiveness of the shock-responsive social protection system. Establishing partnerships through adopting PCD would therefore be beneficial for the Eswatini government because the PCD framework would ensure DRR, CC and FS strategies, plans and interventions are done collaboratively between stakeholders such as government departments, non-governmental organisations (NGOs) and relief agencies (Shah, Ahmmed & Khalid 2022). Furthermore, taking a multiple stakeholder approach can address the coordination aspect of initiatives and response actions taken during CC impact or a disaster (SRSAP 2017).

As was the case with pillar one, for the most part, policy coherence relating to pillar two functions on a conceptual coherence level through the recognition in policy of the need for cross-sectoral cooperation and linkages to address complex developmental problems, such as the nexus. However, there are currently no coherent strategic or institutional mechanisms in place to enforce the integrated management of the nexus in the country, thereby leading to fragmented programme implementation at an operational level. Additionally, financial coherence is also absent in the pillar, with no coordinated resource mobilisation strategy currently in place to facilitate the sustainable funding of nexus-related programmes between government departments, sub-national government, donors and other societal stakeholders.

The following building block addresses and elaborates on the importance of policies, implementation tools and strategic governance actions. This will set the tone for the next section, which looks into building blocks related to Pillar 3.

### Building blocks related to Pillar 3

The last two building blocks address the last pillar, which is to have a set of responsive and adaptive tools to assess and address domestic, transboundary and long-term impacts of policies. Specifically, the section will address policy effects and end with monitoring, reporting and evaluation.

#### Policy effects

According to the OECD (2015), policy effects entail assessing and analysing policy impacts to provide decision-makers with informed evidence on the positive and negative impacts of domestic policies on sustainable development. It is argued that critically analysing and examining the impact of policies and strategies assists in understanding the value, effectiveness and meaningfulness of policies within government. The Government of Eswatini has developed the Public Policy Coordination Unit (PPCU) as a means of coordinating policy formulation, review and monitoring of the policy implementation process. However, the PPCU’s ability to measure policy efficacy from policy formulation to project implementation is severely constrained. This is because the line ministries, which on a political level have higher authority than the PPCU, have been mandated by law to track the efficacy of the implementation of policies and projects. This means that, in reality, the PPCU currently only has the authority to monitor the efficacy of the policy development process and not the actual policy implementation, which is vested in line departments (National Disaster Management Agency [NDMA] 2022). Introducing a PCD approach could aid in remedying the current situation by supporting legal reform, fostering political commitment and stakeholder coordination buy-in (e.g. from line departments) to support the expansion of the mandate of the PPCU to also monitor the efficacy of project implementation. The expansion of the PPCU mandate would further foster the efficiency of DRR, CC and FS strategies and initiatives while coordinating activities and eliminating duplication between responsible line ministries.

#### Monitoring, reporting and evaluation

The building block Monitoring and Evaluation addresses the investigation of policy accountability and outcome delivery (OECD 2023). Specifically, monitoring is regarded as the tool to help government and stakeholders improve operational action and activities and ensure that set strategic goals are met (Mabizela & Zwane 2023). Evaluation, on the other hand, looks at the process of accumulating evidence to determine the value and relevance of an intervention or project with regard to set strategic objectives (Mabizela & Zwane 2023). Smilka (2019) adds that monitoring, reporting and evaluation seek to collect and evaluate information on the impact of policies on sustainable development efforts and report regularly to governing bodies and the public about progress on how policies have been integrated to reach maximum strategic objective realisation. According to Ile, Allen-Ile and Chukuakadibia (2012), it is crucial for governments to analyse evidence collected through policy monitoring mechanisms and to report on the impacts of policies. Monsonís-Payá, Iñigo and Blok (2023) suggest that methods and tools designed for monitoring or evaluation purposes, such as procedures, evaluation assessments and quantitative or qualitative indicators, assist with determining and achieving effective governance. This means that policies could be validated, gaps identified and their value enforced through the implementation of this building block (Mabizela & Zwane 2023; OECD 2018).

In Eswatini, monitoring and evaluation are executed through an existing monitoring and evaluation framework. The monitoring and evaluation framework is disseminated to all government ministries through the deployment of Monitoring and Evaluation officers in every government ministry. This work is not done by Monitoring and Evaluation personnel alone; the PPCU also has to contribute to reviewing as well as monitoring the implementation process of all policies made (SRSAP 2017). However, a major gap that exists with the monitoring mechanisms within Eswatini is that they are currently conducted in silos. For instance, the PPCU, line departments and evaluation officers all conduct monitoring and evaluation processes separately. This leads to the duplication of tasks and monitoring and evaluation reports that are not aligned. Because of the fragmented nature of Monitoring and Evaluation mechanisms within Eswatini, it is difficult to get a holistic picture of the extent of implementation and possible gaps in developmental programmes involving CC, FS and DRR. Policy coherence for development could provide a way to remedy the fragmented monitoring and evaluation landscape through active efforts to establish platforms for various entities involved in monitoring and evaluation in the country to come together and integrate their existing data on the status quo of developmental project implementation to gain a more holistic picture of project successes and gaps. Additionally, the platforms can create a unified approach and policies to guide monitoring and evaluation of developmental programmes in the future.

Pillar 3 differs from the previous two pillars in that there is both existing conceptual and strategic coherence within Eswatini through the establishment of monitoring and evaluation frameworks and the recognition of the importance of assessing policy impacts. Nonetheless, institutional and operational coherence are absent, as responsibility for monitoring and evaluation is fragmented between the PPCU, line ministries and other government officers. This results in the duplication and misalignment of monitoring and evaluation reports that could give insights into the current progress and challenges in implementing nexus-related programmes in the country. As with the other pillars, financial coherence remains absent within this pillar, with a non-unified approach to fund policy monitoring and evaluation or impact assessment across ministries or entities tasked with monitoring and evaluation functions.

An important aspect that constantly emerges in the discussion of the pillars of PCD is the need for programmes and initiatives to be coordinated and integrated across different sectors and levels of governance. This analysis ensures that strategic goals remain the focus and performance is managed. The following section will address the benefits PCD provided to the vertical and horizontal integration of the FS, CC and DRR nexus.

#### Policy coherence for development: Fostering horizontal and vertical policy integration

In addition to the benefits that the various building blocks and pillars of PCD can bring to the management of the CC, FS and DRR nexus in Eswatini, PCD also introduces the need to manage the problem at different spatial scales through the concepts of horizontal and vertical policy integration. According to Gomar, Stringer and Paavola (2014), governance occurs at different levels, creating an overlapping network of actors with different goals and complex power dynamics. Nilsson et al. (2012) refer to vertical governance as decision-making by the central government that extends towards lower levels of government, while horizontal governance refers to decision-making within line ministries or at the same level of government (Carbone 2016; Nilsson et al. 2012).

Horizontal policy coherence relates to minimising the difference in related policy areas within the same government (Mackie 2020). Sandström et al. (2020) echo that horizontal policy coherence aims to bring different government departments in charge of different policy issues together and streamline their policies and programme implementation for improved governance. An example of horizontal policy coherence practice in Eswatini is the advocacy of mainstreaming DRR, CC and FS issues throughout all plans, strategies and initiatives of all government ministries. However, as stated under Section 2.1.4.1, different ministries within central government are currently mandated by law to execute and manage the individual aspects of the DRR, CC and FS nexus independently, rather than in a coordinated fashion. The lack of collaborative efforts towards addressing the nexus leads to government resources being inefficiently spent, duplication of programming and long-term national development policy deliverables not being achieved (Shah et al. 2022). Integrating a PCD approach could assist the government of Eswatini to strengthen horizontal policy coherence by identifying and eliminating existing sectoral overlaps, managing trade-offs and opportunities that exist within the CC, FS and DRR nexus, optimising the use of limited human and financial resources and drawing attention to the lack of alignment within government.

Vertical policy coherence refers to the clear stipulation of mandates, roles, responsibility, consistency, coordination and collaboration across different levels of government (Sandström et al. 2020). Vertical coherence characterises a policy system that exists within different levels of government within the same policy scope (Mackie 2020; OECD 2003). Vertical coherence involves an effort to harmonise policies and strategies from the national, sub-national and eventually local levels (Sandström et al. 2020). For example, in Eswatini, vertical policy coherence is facilitated by the duplication of key departments at the national, regional and local levels. This duplication ideally ensures that development mandates and policies that are formulated at a national level are integrated and implemented up to the local level. Although Eswatini is working towards decentralisation of government and government developmental policy on a policy level, the country is struggling to achieve implementation at operational level.

Enhancing vertical coherence through the integration of a PCD approach will ensure a holistic approach to governance that uses decision-making systems at all levels of government to address the involvement of stakeholders and ensure enhanced government coordination. Policy coherence for development can contribute to greater vertical policy integration by facilitating linkages between policy formulation, decision-making and project implementation at different government levels and ensuring greater alignment. The PCD framework would help Eswatini address the gap that currently exists in the country’s efforts to manage the DRR, FS and CC nexus in a decentralised manner. It would enhance community involvement by promoting and creating community-led governance structures that can assist in setting achievable goals, developing programme activities and managing project activities that contribute to achieving the sustainable management of the DRR, FS and CC nexus.

## Conclusion

The governance of the CC, FS and DRR nexus in Eswatini highlights both progress and persistent challenges. The country has made notable advances in developing a conceptual foundation that recognises resilience, adaptation and vulnerability as guiding principles for managing complex developmental issues such as those presented by the nexus. Yet, it was found that the translation of this conceptual policy coherence into strategic, institutional, operational and financial practice remains weak or absent (Nemakonde et al. 2021; Scott & Ting 2021). It was established that policies are often well formulated but locked into ministerial silos, undermined by weak intergovernmental coordination, limited stakeholder engagement and the absence of integrated funding mechanisms. This results in duplication, inefficiency and a widening gap between national developmental ambitions and local realities related to the management of the CC, FS and DRR nexus in Eswatini. These shortcomings are especially significant in light of Eswatini’s acute socio-economic and environmental vulnerability. With over 70% of the country’s population dependent on subsistence farming, recurrent droughts, floods and heatwaves continually threaten food availability and nutritional security. Environmental shocks intensify chronic poverty and dependency on humanitarian relief, further straining government resources. Despite the proliferation of over a dozen policies and strategies related to managing the nexus, fragmented implementation has yielded little progress towards the 2030 Agenda or national development vision.

Against this backdrop, adopting a PCD framework offers a viable pathway for systemic reform. Policy coherence for development emphasises horizontal and vertical integration across departments and among levels of government, joint planning and implementation responsibility, establishment of integrated governance mechanisms and the fostering of political will to manage policy trade-offs and synergies needed to effectively manage the CC, FS and DRR nexus (OECD 2023; Zembe, Nemakonde & Chipangura 2023). Its application could break down entrenched silos by institutionalising inter-ministerial and multi-stakeholder platforms, clarifying roles and responsibilities and aligning sectoral mandates with shared national objectives. By fostering such linkages, PCD would help consolidate resources and facilitate the development of a unified strategy for managing the CC, FS and DRR nexus. Financial and operational coherence represents additional priorities. Current budget structures reinforce fragmentation with ministries competing for scarce funds rather than pooling resources. Policy coherence for development offers a framework for integrated budget allocation and cross-sectoral funding arrangements that incentivise collaboration rather than competition (Chikezie et al. 2024; Koff et al. 2020). This institutional and financial scaffolding is essential for translating well-articulated policies into meaningful actions during project implementation (Mkhatshwa et al. 2024; Shah et al. 2022). The research also established that current monitoring, evaluation and accountability systems in Eswatini are also fragmented in their implementation. Line ministries, the Public Policy Coordination Unit (PPCU) and evaluation officers produce separate reports that fail to capture the holistic picture of progress in implementing programmes associated with the nexus. Expanding the PPCU’s mandate to include oversight of implementation, coupled with integrated reporting platforms, could strengthen the accountability of all stakeholders involved in managing the nexus and enable evidence-based policy adjustment. Embedded within the broader PCD framework, such a mechanism would reduce duplication, identify gaps and accelerate progress towards sustainable development.

Using the case of Eswatini, this article has illustrated that the development of ambitious policies is insufficient without the deliberate institutionalisation of policy coherence and integrated governance by key governmental and societal stakeholders. By embedding PCD principles into policy-making structures, enhancing coordination across government levels and aligning financial and monitoring systems with long-term strategies, Eswatini can move from conceptual recognition to managing the CC, FS and DRR nexus in an integrated manner to a practical achievement.
